# Supplementary material for: Genome-Wide Identification, Classification, and Expression Analyses of the CsDGAT Gene Family in Cannabis sativa L. and Their Response to Cold Treatment
Source: Int J Mol Sci. 2023 Feb 17;24(4):4078. doi: 10.3390/ijms24044078 (PMC9963917; doi:10.3390/ijms24044078)
Supplement: Supplementary file 1 [file ijms-24-04078-s001.zip › Table S1. The DGAT1, DGAT2 DGAT3 and WSD enzymes used for the phylogenetic analyses.pdf]

Table S1 The DGAT1, DGAT2 DGAT3 and WSD enzymes used for the phylogenetic analyses.

Specie	Taxa terminologies	Gene	Database	Access
Aquilegia coerulea	*Aq*	*DGAT3*	JGI	Aquca_003_00301.1
Arabidopsis lyrata	*Al*	*DGAT3*	JGI	913875
Arabidopsis thaliana	*At*	*DGAT1*	TAIR	AT2G19450
Arabidopsis thaliana	*At*	*DGAT2*	TAIR	AT3G51520
Arabidopsis thaliana	*At*	*DGAT3*	TAIR	AT1G48300
Arabidopsis thaliana	*At*	*WS/DGAT1*	TAIR	AT5G37300.1
Arabidopsis thaliana	*At*	*WS/DGAT6*	TAIR	AT3G49210.1
Arabidopsis thaliana	*At*	*WS/DGAT7*	TAIR	AT5G12420.1
Arachis hypogaea	*Ah*	*DGAT1-1*	NCBI	KC736068
Arachis hypogaea	*Ah*	*DGAT1-2*	NCBI	KC736069
Arachis hypogaea	*Ah*	*DGAT2*	NCBI	AEO11788
Arachis hypogaea	*Ah*	*DGAT3-1*	NCBI	ABW34442
Arachis hypogaea	*Ah*	*DGAT3-2*	NCBI	AAX62735
Arachis hypogaea	*Ah*	*DGAT3-3*	NCBI	KC736067
Arachis hypogaea	*Ah*	*WS/DGAT*	JGI	arahy.Tifrunner.gnm1.ann1.J3RNWS.1
Brachypodium distachyon	*Bradi*	*DGAT1*	JGI	Bradi1g37750.1
Brachypodium distachyon	*Bradi*	*DGAT1*	JGI	Bradi2g33180.1
Brachypodium distachyon	*Bradi*	*DGAT2*	JGI	Bradi1g42650.1
Brachypodium distachyon	*Bradi*	*DGAT2*	JGI	Bradi3g53247.1
Brachypodium distachyon	*Bradi*	*DGAT3*	JGI	Bradi2g36890.1
Brassica juncea	*Bj*	*DGAT1*	NCBI	AAY40784.1
Brassica napus	*Bn*	*DGAT2*	NCBI	ACO90187
Brassica napus	*Bn*	*DGAT1*	NCBI	AAD45536.1

Brassica napus	*Bn*	*DGAT1*	NCBI	AFM31260.1
Brassica napus	*Bn*	*DGAT1*	NCBI	AFM31259.1
Brassica napus	*Bn*	*DGAT1*	NCBI	AFM31262.1
Brassica rapa	*Bra*	*DGAT1*	JGI	Brara.G00164.1
Brassica rapa	*Bra*	*DGAT1*	JGI	Brara.I01120.1
Brassica rapa	*Bra*	*DGAT2*	JGI	Brara.A02083
Brassica rapa	*Bra*	*DGAT2*	JGI	Brara.C04367.1
Brassica rapa	*Bra*	*DGAT3*	JGI	Brara.H00366.1
Brassica rapa	*Bra*	*WS/DGAT*	JGI	Brara.B01454.1.p
Brassica rapa	*Bra*	*WS/DGAT*	JGI	Brara.B00458.1
Brassica rapa	*Bra*	*WS/DGAT*	JGI	Brara.C01989.1
Capsella rubella	*Car*	*DGAT3*	JGI	Carubv10011099m
Carica papaya	*Cp*	*DGAT3*	JGI	evm.model.supercontig_146.13
Chlamydomonas reinhardtii	*Cre*	*DGAT1*	JGI	Cre01g045903.t1.1
Chlamydomonas reinhardtii	*Cre*	*DGAT2*	JGI	Cre06.g299050.t1.2
Chlamydomonas reinhardtii	*Cre*	*DGAT2*	JGI	Cre09.g386912.t1.1
Chlamydomonas reinhardtii	*Cre*	*DGAT2*	JGI	Cre02.g079050.t1.3
Chlamydomonas reinhardtii	*Cre*	*DGAT2*	JGI	Cre03.g205050.t1.2
Chlamydomonas reinhardtii	*Cre*	*DGAT2*	JGI	Cre12.g557750.t1.3
Citrus clementina	*Cic*	*DGAT3*	JGI	Ciclev10020833m
Citrus clementina	*Cic*	*DGAT3*	JGI	Ciclev10005574m
Citrus sinensis	*Csi*	*DGAT3*	JGI	orange1.1g023360m
Citrus sinensis	*Csi*	*DGAT3*	JGI	orange1.1g018470m
Echium pitardii	*Ep*	*DGAT1*	NCBI	ACO55635.1
Eucalyptus grandis	*Eugr*	*DGAT3*	JGI	Eucgr.H04131.1
Euonymus alatus	*Ea*	*DGAT1*	NCBI	AAV31083.1

Fragaria vesca	*Fve*	*DGAT3*	JGI	mrna22793.1-v1.0-hybrid
Glycine max	*Glyma*	*DGAT1*	JGI	Glyma09G065300.1
Glycine max	*Glyma*	*DGAT1*	JGI	Glyma13G106100.1
Glycine max	*Glyma*	*DGAT1*	JGI	Glyma17G053300.1
Glycine max	*Glyma*	*DGAT2*	JGI	Glyma09G195400.1
Glycine max	*Glyma*	*DGAT2*	JGI	Glyma11G088800.1
Glycine max	*Glyma*	*DGAT2*	JGI	Glyma01G156000.1
Glycine max	*Glyma*	*DGAT2*	JGI	Glyma16G115700.1
Glycine max	*Glyma*	*DGAT2*	JGI	Glyma16G115800.1
Glycine max	*Glyma*	*DGAT3*	JGI	Glyma13G118300.1
Glycine max	*Glyma*	*DGAT3*	JGI	Glyma17G041600.1
Glycine max	*Glyma*	*WS/DGAT*	JGI	Glyma.09G196400.1
Glycine max	*Glyma*	*WS/DGAT*	JGI	Glyma.06G291700.1
Glycine max	*Glyma*	*WS/DGAT*	JGI	Glyma.12G114400.1
Gossypium raimondii	*Gorai.*	*DGAT3*	JGI	Gorai.007G116800.1
Jatropha curcas	*Jc*	*DGAT1*	NCBI	ABB84383.1
Lotus japonicus	*Lj*	*DGAT1*	NCBI	AAW51456.1
Malus domestica	*Md*	*DGAT3*	JGI	MDP0000192819
Manihot esculenta	*Manes*	*DGAT3*	JGI	Manes.01G234700
Manihot esculenta	*Manes*	*DGAT3*	JGI	Manes.03G212700
Medicago truncatula	*Medtr*	*DGAT1*	JGI	Medtr2g039940.1
Medicago truncatula	*Medtr*	*DGAT2*	JGI	Medtr5g024990.1
Medicago truncatula	*Medtr*	*DGAT2*	JGI	Medtr8g072550.1
Medicago truncatula	*Medtr*	*DGAT2*	JGI	Medtr8g072540.1
Medicago truncatula	*Medtr*	*DGAT3*	JGI	Medtr4g124080.1
Micromonas pusilla RCC299	*Mp*	*DGAT2*	JGI	EuGene.0500010193

Micromonas pusilla RCC300	*Mp*	*DGAT2*	JGI	EuGene.1000010156
Micromonas pusilla RCC301	*Mp*	*DGAT2*	JGI	fgenesh2_pg.C_Chr_03000495
Micromonas pusilla RCC302	*Mp*	*DGAT2*	JGI	e_gw2.06.451.1
Micromonas pusilla RCC303	*Mp*	*DGAT2*	JGI	est_cluster_kg.
Mimulus guttatus	*Migut*	*DGAT3*	JGI	Migut.H01460.1.p
Nicotiana tabacum	*Nt*	*DGAT1*	NCBI	AAF19345.1
Olea europaea	*Oe*	*DGAT1*	NCBI	AAS01606.1
Oryza sativa	*Os*	*DGAT1*	JGI	Os06g36800.1
Oryza sativa	*Os*	*DGAT1*	JGI	Os05g10810.1
Oryza sativa	*Os*	*DGAT2*	JGI	Os02g48350.1
Oryza sativa	*Os*	*DGAT2*	JGI	Os06g22080.1
Oryza sativa	*Os*	*DGAT3*	JGI	Os05g04620.1
Oryza sativa	*Os*	*WS/DGAT1*	JGI	Os01g48874.1
Oryza sativa	*Os*	*WS/DGAT3*	JGI	Os01g48874.2
Oryza sativa	*Os*	*WS/DGAT2*	JGI	Os05g48260.1
Ostreococcus lucimarinus	*Ol*	*DGAT2*	JGI	eugene.1200010135
Ostreococcus lucimarinus	*Ol*	*DGAT2*	JGI	eugene.1500010069
Ostreococcus lucimarinus	*Ol*	*DGAT2*	JGI	fgenesh1_pm.C_Chr_13000021
Ostreococcus lucimarinus	*Ol*	*DGAT2*	JGI	fgenesh1_pg.C_Chr_8000176
Panicum virgatum	*Pavir*	*DGAT1*	JGI	J36735.1
Panicum virgatum	*Pavir*	*DGAT1*	JGI	J31697.1
Panicum virgatum	*Pavir*	*DGAT1*	JGI	J02054.1
Panicum virgatum	*Pavir*	*DGAT1*	JGI	Da00530.1
Panicum virgatum	*Pavir*	*DGAT2*	JGI	J34441.1
Panicum virgatum	*Pavir*	*DGAT2*	JGI	Ab03087.1
Panicum virgatum	*Pavir*	*DGAT2*	JGI	Da00364.1

Panicum virgatum	*Pavir*	*DGAT2*	JGI	Db01505.1
Perilla frutescens	*Pf*	*DGAT1*	NCBI	AAG23696.1
Phaseolus vulgaris	*Phvul*	*DGAT1*	JGI	Phvul009G230700.1
Phaseolus vulgaris	*Phvul*	*DGAT1*	JGI	Phvul003G134900.1
Phaseolus vulgaris	*Phvul*	*DGAT2*	JGI	Phvul002G119500.1
Phaseolus vulgaris	*Phvul*	*DGAT2*	JGI	Phvul003G272500.1
Phaseolus vulgaris	*Phvul*	*DGAT2*	JGI	Phvul003G272600.1
Phaseolus vulgaris	*Phvul*	*DGAT3*	JGI	Phvul003G123000.1
Physcomitrella patens	*Phpat*	*DGAT1*	JGI	Pp3c15_23830
Physcomitrella patens	*Phpat*	*DGAT1*	JGI	Pp3c9_24440
Physcomitrella patens	*Phpat*	*DGAT2*	JGI	Pp3c22_20760
Physcomitrella patens	*Phpat*	*DGAT2*	JGI	Pp3c14_20110
Populus trichocarpa	*Potri*	*DGAT1*	JGI	Potri018G066100.1
Populus trichocarpa	*Potri*	*DGAT1*	JGI	Potri006G147600.1
Populus trichocarpa	*Potri*	*DGAT2*	JGI	Potri011G145900.1
Populus trichocarpa	*Potri*	*DGAT3*	JGI	Potri002G187300.1
Populus trichocarpa	*Potri*	*DGAT3*	JGI	Potri010G003200.1
Ricinus communis	*Rc*	*DGAT1*	NCBI	29912.t000099
Ricinus communis	*Rc*	*DGAT2*	JGI	29682.t000014
Ricinus communis	*Rc*	*DGAT3*	JGI	29889.t000177
Selaginella moellendorffii	*Sm*	*DGAT1*	JGI	Sm_404425
Selaginella moellendorffii	*Sm*	*DGAT1*	JGI	Sm_81638
Selaginella moellendorffii	*Sm*	*DGAT2*	JGI	Sm_96204
Setaria italica	*Si*	*DGAT1*	JGI	Si006298m.g
Setaria italica	*Si*	*DGAT1*	JGI	Si021762m.g
Setaria italica	*Si*	*DGAT2*	JGI	Si017737m.g

Setaria italica	*Si*	*DGAT2*	JGI	Si006465m.g
Solanum lycopersicum	*Soly*	*DGAT3*	JGI	Solyc12g098850.1.1
Solanum tuberosum	*St*	*DGAT3*	JGI	PGSC0003DMP400008124
Sorghum bicolor	*Sobic*	*DGAT1*	JGI	Sobic010G170000.1
Sorghum bicolor	*Sobic*	*DGAT1*	JGI	Sobic009G072700
Sorghum bicolor	*Sobic*	*DGAT2*	JGI	Sobic010G134400.1
Sorghum bicolor	*Sobic*	*DGAT2*	JGI	Sobic004G261900.1
Sorghum bicolor	*Sobic*	*DGAT2*	JGI	Sobic.008G049000.3
Sorghum bicolor	*Sobic*	*DGAT3*	JGI	Sobic009G034600.1
Sorghum bicolor	*Sobic*	*WS/DGAT*	JGI	SbiRTX430.03G334100.1.p
Theobroma cacao	*Thecc*	*DGAT3*	JGI	Thecc1EG004941t1
Tropaeolum majus	*Tm*	*DGAT1*	NCBI	AAM03340.2
Vernicia fordii	*Vf*	*DGAT1*	NCBI	ABC94472.1
Vernonia galamensis	*Vg*	*DGAT1*	NCBI	ABV21945.1
Vitis vinifera	*Vv*	*DGAT3*	JGI	GSVIVT01017699001
Vitis vinifera	*Vv*	*DGAT1*	NCBI	XP_002279345.1
Volvox carteri	*Vocar*	*DGAT1*	JGI	Vocar20008498m.g
Volvox carteri	*Vocar*	*DGAT2*	JGI	Vocar.0001s0245
Volvox carteri	*Vocar*	*DGAT2*	JGI	Vocar.0008s0353.1
Volvox carteri	*Vocar*	*DGAT2*	JGI	Vocar.0047s0035
Zea mays	*Zm*	*DGAT1*	JGI	GRMZM2G130749
Zea mays	*Zm*	*DGAT1*	JGI	GRMZM2G169089
Zea mays	*Zm*	*DGAT2*	JGI	GRMZM2G042356
Zea mays	*Zm*	*DGAT2*	JGI	GRMZM2G050641
Zea mays	*Zm*	*DGAT3*	JGI	GRMZM2G122943_T01
Cannabis sativa	*Cs*	*DGAT1*	NCBI	XP_030487910.1

Cannabis sativa	*Cs*	*DGAT2*	NCBI	XP_030486482.1
Cannabis sativa	*Cs*	*DGAT3*	NCBI	XP_030507626.1
Cannabis sativa	*Cs*	*WS/DGAT1.1*	NCBI	XP_030501890.1
Cannabis sativa	*Cs*	*WS/DGAT1.2*	NCBI	XP_030492184.1
Cannabis sativa	*Cs*	*WS/DGAT1.3*	NCBI	XP_030486720.1
Cannabis sativa	*Cs*	*WS/DGAT1.4*	NCBI	XP_030488196.1
Cannabis sativa	*Cs*	*WS/DGAT1.5*	NCBI	XP_030503296.1
Cannabis sativa	*Cs*	*WS/DGAT1.6*	NCBI	XP_030492330.1
Cannabis sativa	*Cs*	*WS/DGAT1.7*	NCBI	XP_030486282.1
