# Supplementary material for: Genome-Wide Identification, Classification, and Expression Analyses of the CsDGAT Gene Family in Cannabis sativa L. and Their Response to Cold Treatment
Source: Int J Mol Sci. 2023 Feb 17;24(4):4078. doi: 10.3390/ijms24044078 (PMC9963917; doi:10.3390/ijms24044078)
Supplement: Supplementary file 1 [file ijms-24-04078-s001.zip › Table S3. Primers used for QRT.pdf]

Table S3. Primers used for subcellular localization

Gene	Sequence
CsDGAT1F	cagtCGTCTCacaacatggcgatttcagattcgcc
CsDGAT1R	cagtCGTCTCatacattcatttgtccctttcggt
CsDGAT2F	cagtCGTCTCacaacatggtgctggataatcagaa
CsDGAT2R	cagtCGTCTCatacaaaggactttcaactcaaggt
CsDGAT3F	cagtCGTCTCacaacatggaggtttccgggttccttcc
CsDGAT3R	cagtCGTCTCatacaaactgatgcagccaaccccaaatcattg
CsWSD1.1F	cagtGGTCTCacaacatgagtgggggcggcgatg
CsWSD1.1R	cagtGGTCTCatacagacaatttccttgttcatcctctgctcaagaatag
CsWSD1.4F	cagtCGTCTCacaacatggaacctgaagaaggtagtgttcgac
CsDWSD1.4R	cagtCGTCTCatacattccataacagtagtttttgactgatatttctcaactacag
