# Supplementary material for: Genome-Wide Identification, Classification, and Expression Analyses of the CsDGAT Gene Family in Cannabis sativa L. and Their Response to Cold Treatment
Source: Int J Mol Sci. 2023 Feb 17;24(4):4078. doi: 10.3390/ijms24044078 (PMC9963917; doi:10.3390/ijms24044078)
Supplement: Supplementary file 1 [file ijms-24-04078-s001.zip › Table S4. Expression levels of CsDGAT genes in hemp different varieties based on transcriptome expression data.pdf]

Table S4. Expression levels of *CsDGAT* genes in hemp different varieties based on transcriptome expression data

Gene name	Varieties								
	Blackberry Kush	Black Lime	Cherry Chem	Canna Tsu	Mama Thai	Sour Diesel	Terple	Valley Fire	White Cookies
DGAT1	23.69757881	26.93819867	11.21502566	28.26532282	21.92621501	51.75651997	21.33940707	43.9993756	27.05278734
DGAT2	4.511627871	6.624751987	6.451530232	6.604363599	5.571167481	9.212268791	7.32359415	9.459155926	9.057085507
DGAT3	28.27914902	30.64001309	24.25456632	36.54814976	50.32785399	30.15424747	24.17681369	21.80001972	18.20999722
CsWSD1.1	8.326863439	2.731753408	2.262103122	1.247117667	2.95032925	1.87285439	2.802380928	4.503905092	2.367005506
CsWSD1.2	6.383866708	16.60134237	54.30921524	12.33719441	19.01090341	39.06888234	15.77478254	21.22419218	4.156155457
CsWSD1.3	0.501095007	0.787279626	0.15357504	0.13579095	0.260712111	0.120387026	0.669912887	2.914963388	0.246703323
CsWSD1.4	1.324611775	0	0.157428896	0	0.302605475	0.074676809	0	0.201773964	0
CsWSD1.5	0.423116988	0.08915882	1.314156333	0.07708693	0.481005642	0.331927075	0.074059543	0.240687226	0.068265581
CsWSD1.6	0.092751487	0.087268453	0.014125824	0.132150267	1.748604848	0.036521048	0.458927793	0.39294317	0.013562445
CsWSD1.7	16.95695012	12.87896039	1.468791915	1.86371715	13.76718651	2.441460267	9.183908064	9.45417112	2.078445306
