# Supplementary material for: Genome-Wide Identification, Classification, and Expression Analyses of the CsDGAT Gene Family in Cannabis sativa L. and Their Response to Cold Treatment
Source: Int J Mol Sci. 2023 Feb 17;24(4):4078. doi: 10.3390/ijms24044078 (PMC9963917; doi:10.3390/ijms24044078)
Supplement: Supplementary file 1 [file ijms-24-04078-s001.zip › Table S5. Expression levels of CsDGAT genes in hemp different tissues based on transcriptome expression data.pdf]

Table S5. Expression levels of *CsDGAT* genes in hemp different tissues based on transcriptome expression data

**Gene name**	**Tissuees**				
	Root	Stem	Leaf	Flower	Seed
CsDGAT1	16.68989555	14.91194542	9.965772239	18.92905146	14.11570987
CsDGAT2	5.658029959	6.093273905	6.135377377	12.40178864	11.57350379
CsDGAT3	50.55683251	33.14767671	48.04105068	24.53482181	29.58781898
CsWSD1.1	27.88154874	22.8072075	26.3728708	7.211990603	6.660250025
CsWSD1.2	0.138871553	1.567880271	13.76138659	90.63111827	18.56183888
CsWSD1.3	0.507208123	36.02398901	62.8098018	7.670687629	0.572389026
CsWSD1.4	0.010039621	0.420516904	0.009317286	1.920944455	25.37958634
CsWSD1.5	0.072369898	0.68757218	0.084281228	7.163574358	0.889544988
CsWSD1.6	1.156807903	0.178405022	4.131008467	2.856281493	3.166763871
CsWSD1.7	25.82511061	59.49612718	219.6431739	7.067046092	6.495545026
