# Supplementary material for: Genome-Wide Identification, Classification, and Expression Analyses of the CsDGAT Gene Family in Cannabis sativa L. and Their Response to Cold Treatment
Source: Int J Mol Sci. 2023 Feb 17;24(4):4078. doi: 10.3390/ijms24044078 (PMC9963917; doi:10.3390/ijms24044078)
Supplement: Supplementary file 1 [file ijms-24-04078-s001.zip › Table S6. Expression levels of CsDGAT genes in developing seeds of hemp at different growth stages after fertilization.pdf]

Table S6. Expression levels of *CsDGAT* genes in developing seeds of hemp at different growth stages after fertilization

Gene name	Growth stages after fertilization (days)		
	10 d	20 d	27 d
CsDGAT1	14.0056	8.6023	15.4813
CsDGAT2	20.0175	16.7321	17.6489
CsDGAT3	41.836	24.8968	52.4468
CsWSD1.1	7.2513	6.5081	6.8826
CsWSD1.2	11.5908	13.7041	14.8692
CsWSD1.3	0.1409	0.8141	0.5194
CsWSD1.4	37.0738	63.1163	82.7452
CsWSD1.5	1.35	0.1651	0.1843
CsWSD1.6	2.2992	1.5679	5.8092
CsWSD1.7	9.0425	8.9866	8.26
