# Supplementary figures and images for: Genome-Wide Identification, Classification, and Expression Analyses of the CsDGAT Gene Family in Cannabis sativa L. and Their Response to Cold Treatment
Source: Int J Mol Sci. 2023 Feb 17;24(4):4078. doi: 10.3390/ijms24044078 (PMC9963917; doi:10.3390/ijms24044078)

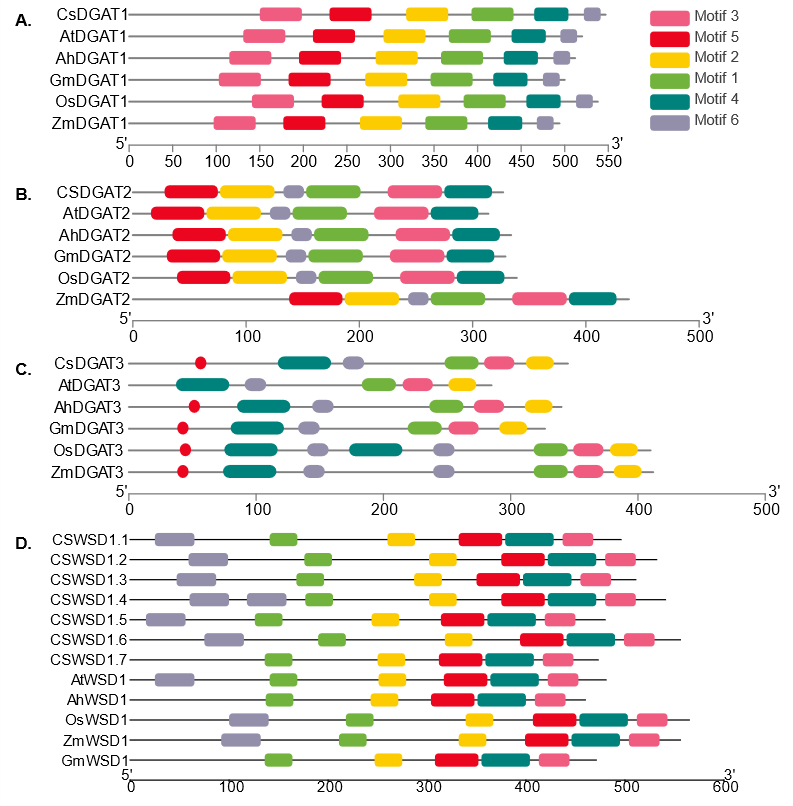

Supplement: Supplementary file 1 [file ijms-24-04078-s001.zip › Figure S1.tif]

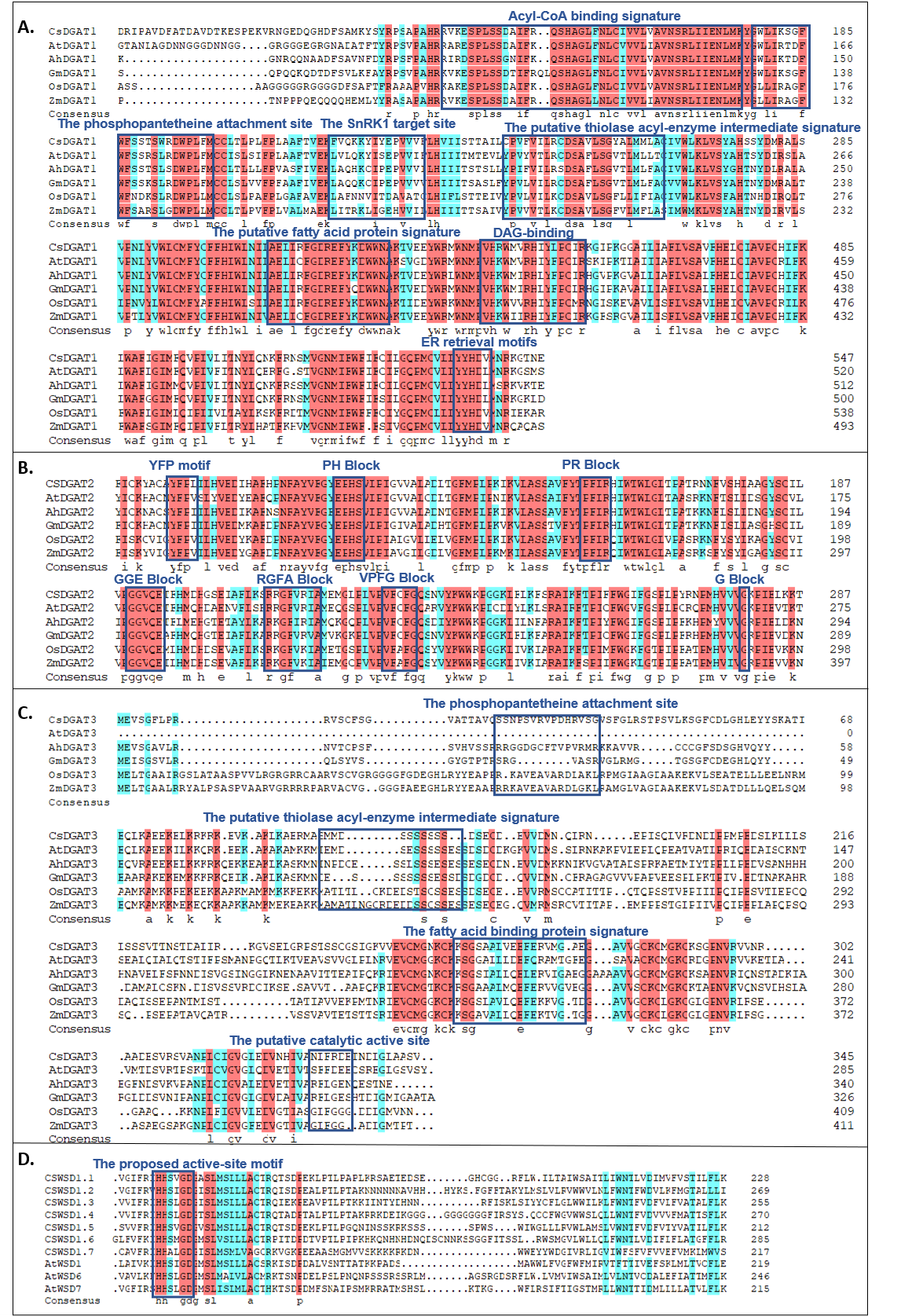

Supplement: Supplementary file 1 [file ijms-24-04078-s001.zip › Figure S2.tif]

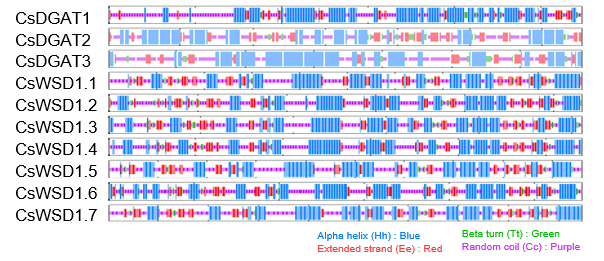

Supplement: Supplementary file 1 [file ijms-24-04078-s001.zip › Figure S3.tif]

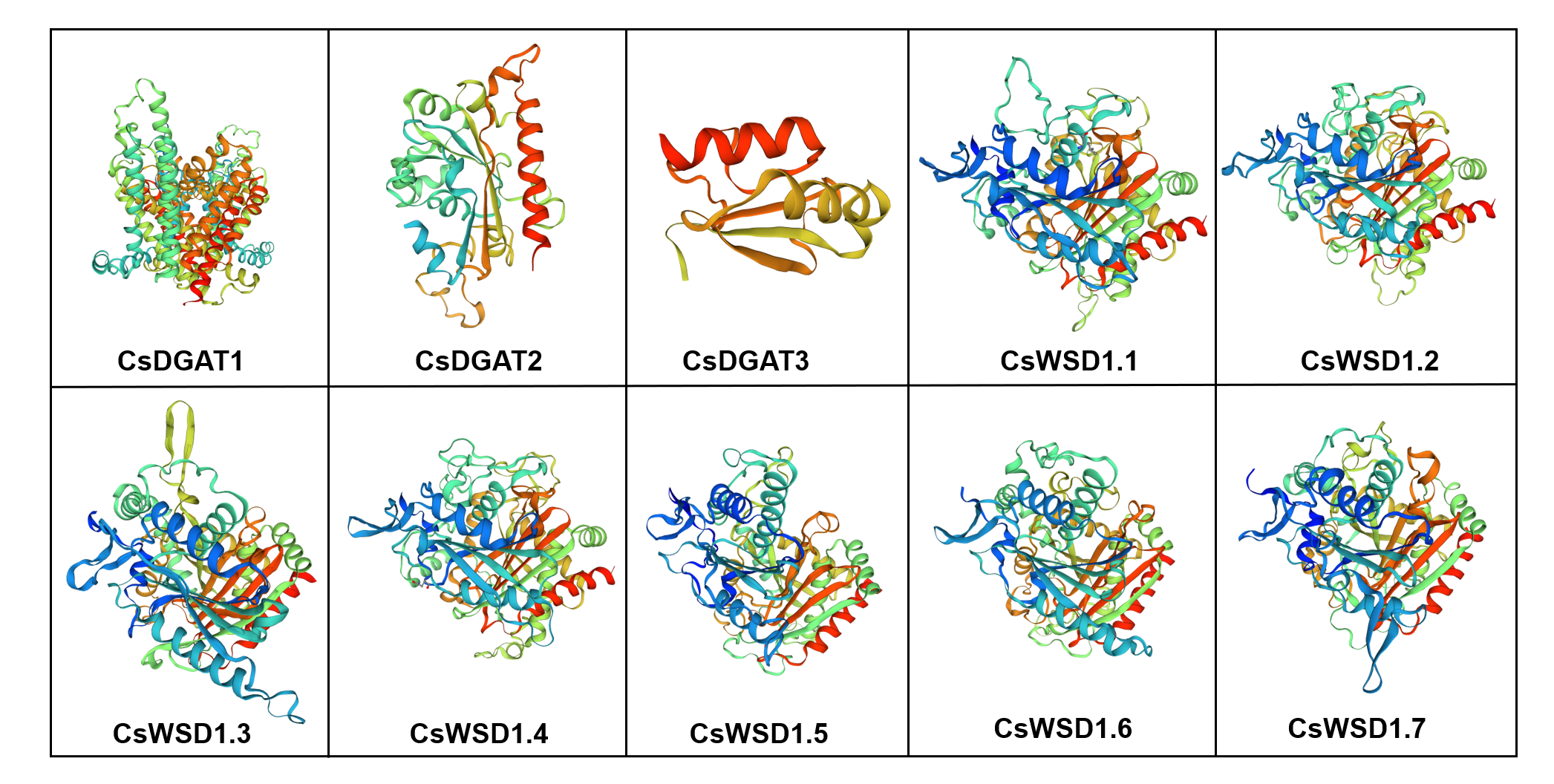

Supplement: Supplementary file 1 [file ijms-24-04078-s001.zip › Figure S4.tif]

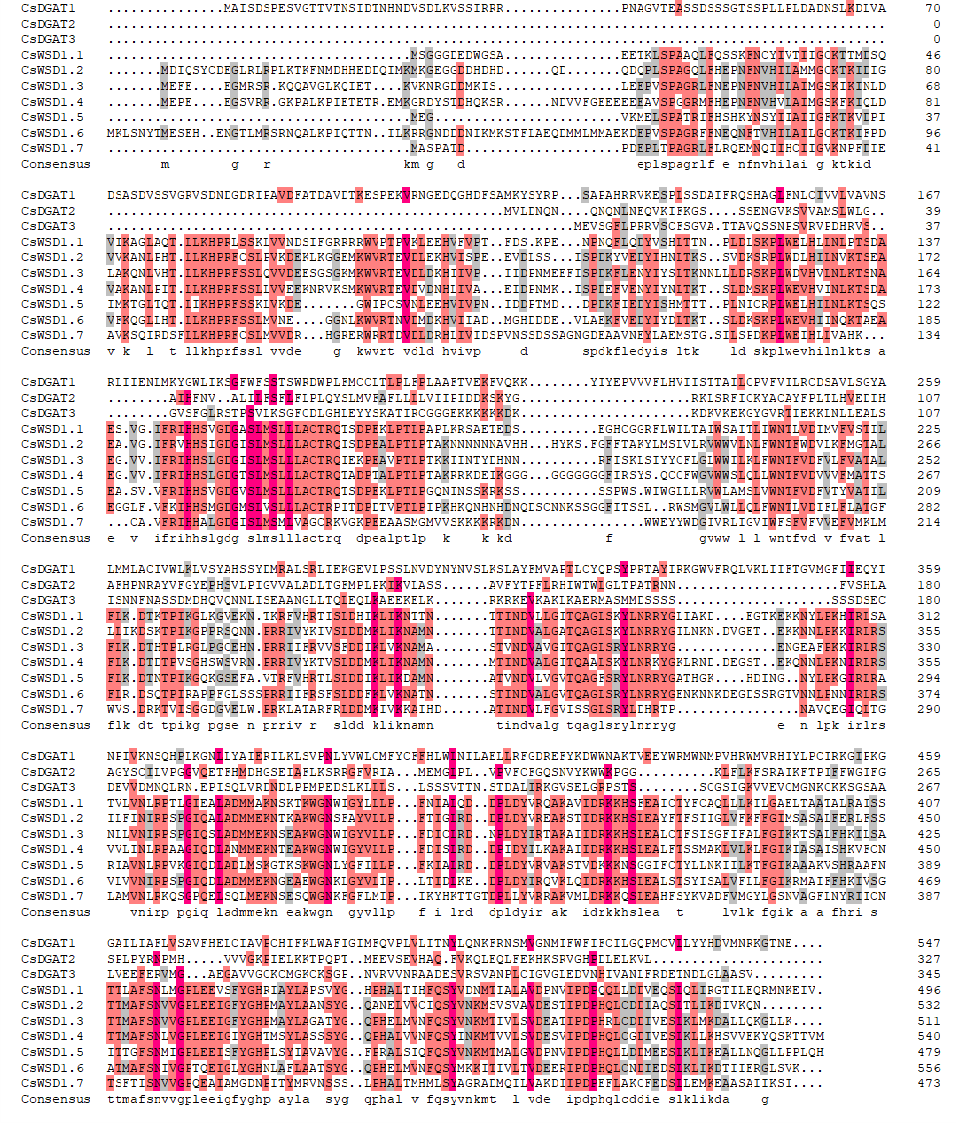

Supplement: Supplementary file 1 [file ijms-24-04078-s001.zip › Figure S5.tif]
